# Depth Profile of Nitrifying Archaeal and Bacterial Communities in the Remote Oligotrophic Waters of the North Pacific

**DOI:** 10.3389/fmicb.2021.624071

**Published:** 2021-02-23

**Authors:** Miguel Semedo, Eva Lopes, Mafalda S. Baptista, Ainhoa Oller-Ruiz, Javier Gilabert, Maria Paola Tomasino, Catarina Magalhães

**Affiliations:** ^1^Interdisciplinary Centre of Marine and Environmental Research (CIIMAR), University of Porto, Matosinhos, Portugal; ^2^Faculty of Sciences, University of Porto, Porto, Portugal; ^3^International Centre for Terrestrial Antarctic Research, University of Waikato, Hamilton, New Zealand; ^4^Department of Chemical & Environmental Engineering, Universidad Politécnica de Cartagena (UPCT), Cartagena, Spain; ^5^School of Science, Faculty of Science and Engineering, University of Waikato, Hamilton, New Zealand

**Keywords:** nitrification, thaumarchaeota, AOA, NOB, Pacific Subtropical Front

## Abstract

Nitrification is a vital ecosystem function in the open ocean that regenerates inorganic nitrogen and promotes primary production. Recent studies have shown that the ecology and physiology of nitrifying organisms is more complex than previously postulated. The distribution of these organisms in the remote oligotrophic ocean and their interactions with the physicochemical environment are relatively understudied. In this work, we aimed to evaluate the depth profile of nitrifying archaea and bacteria in the Eastern North Pacific Subtropical Front, an area with limited biological surveys but with intense trophic transferences and physicochemical gradients. Furthermore, we investigated the dominant physicochemical and biological relationships within and between ammonia-oxidizing archaea (AOA), ammonia-oxidizing bacteria (AOB), and nitrite-oxidizing bacteria (NOB) as well as with the overall prokaryotic community. We used a 16S rRNA gene sequencing approach to identify and characterize the nitrifying groups within the first 500 m of the water column and to analyze their abiotic and biotic interactions. The water column was characterized mainly by two contrasting environments, warm O_2_-rich surface waters with low dissolved inorganic nitrogen (DIN) and a cold O_2_-deficient mesopelagic layer with high concentrations of nitrate (NO_3_^–^). Thaumarcheotal AOA and bacterial NOB were highly abundant below the deep chlorophyll maximum (DCM) and in the mesopelagic. In the mesopelagic, AOA and NOB represented up to 25 and 3% of the total prokaryotic community, respectively. Interestingly, the AOA community in the mesopelagic was dominated by unclassified genera that may constitute a novel group of AOA highly adapted to the conditions observed at those depths. Several of these unclassified amplicon sequence variants (ASVs) were positively correlated with NO_3_^–^ concentrations and negatively correlated with temperature and O_2_, whereas known thaumarcheotal genera exhibited the opposite behavior. Additionally, we found a large network of positive interactions within and between putative nitrifying ASVs and other prokaryotic groups, including 13230 significant correlations and 23 sub-communities of AOA, AOB, NOB, irrespective of their taxonomic classification. This study provides new insights into our understanding of the roles that AOA may play in recycling inorganic nitrogen in the oligotrophic ocean, with potential consequences to primary production in these remote ecosystems.

## Introduction

Nitrogen (N) is an essential element of important biomolecules, such as amino acids, chlorophyll, ATP, and nucleic acids, with a crucial role in regulating ecosystem productivity. In the oligotrophic open ocean, fixed nitrogen is often the growth-limiting nutrient for photosynthetic organisms such as algae and marine bacteria (Tyrrell, 1999). In these environments, pelagic bacteria and archaea can also use the oxidation of inorganic N as their main energy source, in a series of reactions within the nitrification process, performed by ammonia-oxidizing bacteria (AOB) and ammonia-oxidizing archaea (AOA), nitrite-oxidizing bacteria (NOB), and comammox organisms (Winogradsky, 1890; Könneke et al., 2005; Daims et al., 2015; van Kessel et al., 2015).

Until recently, it has been accepted that these nitrifying organisms gain energy in the two-step oxidation of ammonia (NH_3_) to nitrite (NO_2_^–^). In the first step, NH_3_ is oxidized to hydroxylamine (NH_2_OH), by the ammonia monooxygenase (AMO) and in the second step NH_2_OH is oxidized into NO_2_^–^, by hydroxylamine oxidoreductase (HAO). However, recent discoveries proposed a new model for AOB ammonia oxidation where NH_2_OH and also NO are produced as intermediates of these reactions (Caranto and Lancaster, 2017). A HAO homolog remains to be discovered for AOA, thus it is still unclear if archaeal AMO catalyzes the same reactions described for AOB (Lehtovirta-Morley, 2018). While archaea ammonia oxidation pathway is still not resolved, the models that have been developed suggest that this oxidation releases two net electrons that can enter the respiratory chain in both AOA and AOB (Lehtovirta-Morley, 2018).

Ammonia-oxidizing bacteria are rather restricted in their phylogeny and only found in the γ-Proteobacteria and in the formerly β-Proteobacteria, now Betaproteobacteriales (Zehr and Ward, 2002). AOA are only found in the Thaumarchaeota phylum, formerly known as Crenarchaeota (Beman et al., 2010). A recent comparison of whole genome sequences, however, suggests the revision of AOA to the class Nitrososphaeria within the Thermoproteota phylum (Parks et al., 2020). NOB can be found in α-Proteobacteria and the Nitrospirae phylum, but species assigned to the recently proposed Nitrospinae phylum are the dominant NOB in the marine environment (Luecker et al., 2013; Levipan et al., 2014; Sun et al., 2019). The phylogeny of marine nitrifying organisms has been extensively studied and constrained by previous works using nitrification genes sequencing as well as by shotgun metagenomics, supporting the use of 16S-derived phylogeny to infer nitrifier populations (Kowalchuk and Stephen, 2001; Santoro et al., 2010; Sun et al., 2019; Zhong et al., 2020).

In the open ocean, nitrification is a key process for nitrate-based new production and it is partly responsible for the nitrate standing stock in the subeuphotic zone (Zehr and Ward, 2002; Mincer et al., 2007). Nitrifying organisms in these environments occur mainly in the bottom of the euphotic zone, where they can avoid light inhibition and competition by phototropic phytoplankton for fixed N (Pajares and Ramos, 2019). With depth, light becomes limited and nitrifiers can thrive and dominate NH_4_^+^ consumption. The physiochemical environment also has a significant influence in the relative distribution of the different groups of nitrifying organisms in the water column. Thaumarchaeotal AOA, for instance, can make up more than 30% of the total marine picoplankton below the photic zone (Karner et al., 2001) and tend to predominate over AOB in oligotrophic environments due to their higher affinity for NH_4_^+^ (Martens-Habbena et al., 2009; Horak et al., 2013; Kits et al., 2017). AOA are also better adapted to low O_2_ conditions than AOB, which makes them highly abundant in vast oxygen minimum zones of the open ocean (Holtappels et al., 2011; Hollibaugh, 2017). On the other hand, AOA are reported to be more sensitive to light than AOB which, in turn, makes them more vulnerable in the upper layers of the water column (Merbt et al., 2012). Despite all cultured NOB being obligate aerobes, abundant and highly active NOBs can also be found in O_2_-poor waters (Sun et al., 2017, 2019).

Nitrifying organisms are essential to generate accessible forms of N that support primary production below the euphotic zone. Notwithstanding the current knowledge about their ecology and physiology, the interactions between the different groups of nitrifying organisms and with the surrounding environment are still relatively unknown. Especially in the remote oligotrophic ocean due to the inherent limitations accessing the planktonic communities in these areas. Improved knowledge of the dynamics and physicochemical constraints of nitrifying archaea and bacteria is thus crucial for a better understanding of current and future ocean functioning. The goal of this study is to evaluate the depth profile of nitrifying archaea and bacteria in the Eastern North Pacific Subtropical Front, an area with limited biological surveys but with intense trophic transferences and physicochemical gradients (Olson et al., 1994; Sow et al., 2020). Additionally, we aimed to determine the dominant physicochemical and biological relationships within and between the two prokaryotic groups of nitrifying organisms, thaumarchaeotal AOA and nitrifying bacteria (AOB and NOB), as well as with the overall prokaryotic community. We used 16S rRNA gene high-throughput sequencing and physicochemical analyses to identify and characterize the nitrifying groups present within the first 500 m of the water column as well as to describe their interactions with the abiotic environment and among taxa.

## Materials and Methods

### Site Description and Water Sampling

**FIGURE 1:**
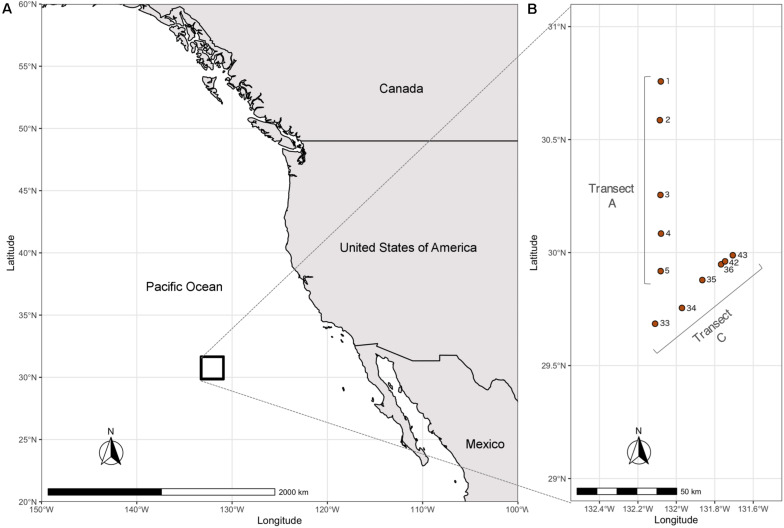
Map of the sampling area and sampling sites. **(A)** Map of North America and Pacific Ocean with sampling area denoted by a black rectangle. **(B)** Sampling sites identified by cast number (information on casts and corresponding samples is detailed in Table 1).

Water column samples were collected in two different transects along the Eastern North Pacific Subtropical Front (ENPSF), 1000 nautical miles off the coast of Southern California, on board of the Schmidt Ocean Institute (SOI) research vessel “Falkor” (Figure 1). The sampled area presents an average bottom depth of around 4500 m. A total of 31 samples were collected in the top 500 m of the water column with a Rosette multi-sampler, between the 1st and 14th of June, 2018 (Table 1). Sea water samples of 3.75 L were filtered with a Sterivex^®^ filter (0.2 μm pore size) for microplankton analysis. The collection filters were stored on board at −80°C and transported in dry ice to CIIMAR for later DNA extraction. The detailed methodology can be found in de Sousa et al. (2019). Samples were classified according to their depth and *in situ* chlorophyll concentrations. Four different depth layers were used in this study: surface (3–5 m, *n* = 11), deep chlorophyll maximum (DCM), (107–130 m, *n* = 11), below DCM (175–200 m, *n* = 4), and mesopelagic (500 m, *n* = 5). The DCM depths observed in this study were similar to the DCM depths previously observed in the Pacific Ocean (Letelier et al., 2004; Sauzède et al., 2020).

**TABLE 1:** Geospatial description of samples used in this study.

Depth Layer	Transect	Sample ID	Cast	Depth (m)	Latitude	Longitude
Surface (3–5 m)	A	4A	1	5	30.586	−132.086
		8A	2	5	29.918	−132.082
		12A	3	5	30.084	−132.080
		16A	4	5	30.255	−132.083
		20A	5	5	30.758	−132.081
	C	53A	33	3	29.878	−131.865
		55A	34	3	29.948	−131.767
		57A	35	3	29.961	−131.746
		59A	36	3	29.988	−131.706
		71A	42	3	29.685	−132.111
		73A	43	3	29.755	−131.970
DCM^*^ (107–130 m)	A	3A	1	120	30.586	−132.086
		7A	2	110	29.918	−132.082
		11A	3	108	30.084	−132.080
		15A	4	122	30.255	−132.083
		18A	5	130	30.758	−132.081
	C	52A	33	123	29.878	−131.865
		54A	34	115	29.948	−131.767
		56A	35	115	29.961	−131.746
		58A	36	115	29.988	−131.706
		70A	42	107	29.685	−132.111
		72A	43	127	29.755	−131.970
Below DCM (175–200 m)	A	2A	1	195	30.586	−132.086
		6A	2	200	29.918	−132.082
		14A	4	175	30.255	−132.083
		10A	3	180	30.084	−132.080
Mesopelagic (500 m)	A	1A	1	500	30.586	−132.086
		5A	2	500	29.918	−132.082
		9A	3	500	30.084	−132.080
		13A	4	500	30.255	−132.083
		17A	5	500	30.758	−132.081

*DCM, Deep Chlorophyll Maximum.

### Physicochemical Parameters

Physicochemical properties of the collected water samples were obtained *in situ* with a Seabird SBE 9 Plus conductivity-temperature-depth (CTD) profiler, deployed with the Rosette. Conductivity (mS/cm), temperature (°C), depth (m), salinity (PSU), oxygen (ml/L), turbidity (NTU), and fluorescence (mg/m^3^) were measured simultaneously in each cast and the complete results from the CTD dataset are publicly available in PANGAEA international archive^1^. Additionally, seawater samples were collected for the quantification of inorganic nitrogen, namely, ammonium (NH_4_^+^), nitrite (NO_2_^–^), and nitrate (NO_3_^–^), at the stations and depths where microplankton samples were collected. These samples were also stored onboard at −80°C. Upon arrival to shore, nutrient samples were transported in dry ice to Technical University of Cartagena, Spain, to be analyzed using a SEAL AA3-HD continuous flow autoanalyzer according to previously described methodology (Strickland and Parsons, 1977; Gordon et al., 1993).

### DNA Extraction and Amplicon Sequencing

Planktonic DNA was extracted from the Sterivex filters using the DNeasy^®^ PowerWater^®^ Sterivex DNA Isolation Kit protocol (Qiagen), following manufacturer’s instructions. The 16S rRNA gene was amplified with the degenerate primer pair 515YF (5′–GTGYCAGCMGCCGCGGTAA–3′) and Y926R-jed (5′–CCGYCAATTYMTTTRAGTTT–3′), targeting the hypervariable V4–V5 region (Caporaso et al., 2011, 2012; Apprill et al., 2015; Parada et al., 2016). This primer set has a broad spectrum of diversity, including the Crenarchaeota/Thaumarchaeota phylum (degeneracy at 515YF), as well as the freshwater and marine clade SAR11 (Alphaproteobacterial class, degeneracy at Y926R-jed) (Apprill et al., 2015; Parada et al., 2016).

The initial PCR reaction included 12.5 ng of template DNA in a total volume of 25 μL. The PCR protocol involved a 3 min denaturation step, followed by 25 cycles of 98°C for 20 s, 60°C for 30 s, and 72°C for 30 s, and, finally, an extension stage at 72°C for 5 min. A second PCR reaction was performed to add indexes and sequencing adapters to the target region, according to manufacturer’s recommendations (Illumina, 2013). Negative controls without template were included in all PCR reactions. Lastly, PCR products were one-step purified and normalized using SequalPrep 96-well plate kit (Thermo Fisher Scientific, Waltham, MA, United States), pooled, and pair-end sequenced in the Illumina MiSeq^®^ sequencer using 2 × 300 bp with the V3 chemistry, according to manufacturer instructions (Illumina, San Diego, CA, United States) at Genoinseq (Cantanhede, Portugal). The results from this 16S amplicon sequencing are publicly available in ENA-EMBL archive with the project accession number PRJEB32783.

### Bioinformatic Analysis

The raw FASTQ files obtained with Illumina MiSeq sequencing were trimmed for primer removal using “cutadapt” v.1.16 and imported into R (version 3.5.1) using “DADA2” package v.1.10.1 (Callahan et al., 2016a). Sample filtering, trimming, error rates learning, dereplication, and amplicon sequence variant (ASV) inference were performed with default settings. Chimeras were removed with the *removeBimeraDenovo* function using the method “consensus.” Taxonomy was assigned with the native implementation of the naive Bayesian classifier and a DADA2-formatted reference database for the SILVA v128 database (Quast et al., 2013). Additionally, for disambiguation, taxonomy was further assigned with the GTDB database (Parks et al., 2018) and with the “DECIPHER” package v.2.10.2 (Wright, 2016) using the IDTAXA classifier (Murali et al., 2018) and the modified training sets provided, namely GTDB 16S (revision 95) and SILVA SSU (v138). These pre-processing steps resulted in 6262 ASVs found, with a median number of 43408 reads per sample (16004–71932), corresponding to 60.1% of the initial number of the sequences (Supplementary Table 1). Taxonomy filtering was performed by removing eukaryotic, mitochondrial, and chloroplast sequences (426 ASVs removed). Relative abundances of each ASV per sample were calculated in the filtered dataset by dividing the absolute abundance (counts) of each ASV by the sum of counts of all ASVs.

To explore the nitrifying communities, taxa were categorized into guilds based on the presence of genes that code for proteins in the pathways of nitrification. These guilds were identified at different taxonomic levels to mitigate the effects of unclassified sequences: Thaumarchaeota, Nitrospinae, Nitrospirae, and Nitrospinota were selected at the phylum level; Nitrosomonadaceae and Nitrosococcaceae at the family level; and *Nitrosococcus*, *Nitrospirae*, *Nitrobacter*, *Candidatus Nitrotoga*, *Nitrotoga*, *Nitrospina*, *Nitrococcus*, *Nitrolancea*, *Candidatus Nitromaritima*, and *Nitromaritima* at the genus level. This taxonomic selection resulted in a final number of 302 putative nitrifying ASVs.

To estimate species richness and α-diversity of selected nitrifying communities, Chao and Shannon indexes were calculated, respectively. β-diversity among these communities was evaluated using the Bray-Curtis dissimilarity calculator and a principal coordinate analysis (PCoA). Due to the presence of samples without any thaumarcheotal or nitrifying bacterial ASVs, a mock ASV with an abundance of 0.0001 was added to all samples. Significant effects of depth in community dissimilarity were tested by multivariate permutational ANOVA (PERMANOVA) using the adonis function of the vegan package in R (Oksanen et al., 2017). Multivariate homogeneity of group dispersion was evaluated using the betadisper function of the same R package. To normalize the α- and β-diversity estimates (based on absolute abundances), the original samples (before taxonomic selection of nitrifying groups) were randomly subsampled to the lowest number of sequences (*n* = 16004 sequences per sample). These estimates were calculated using the phyloseq package in R (McMurdie and Holmes, 2013).

A neighbor-joining phylogenetic tree was constructed with all 6262 ASVs found in these samples using the decipher (Wright, 2016) and phangorn (Schliep, 2011) packages in R according to the workflow provided by Callahan et al. (2016b). The phylogenetic tree of thaumarcheotal ASVs was then visualized and annotated in iTOL v. 5.6.3. (Letunic and Bork, 2019).

### Statistical Analysis

Differences in the α-diversity estimators between the different depth groups (surface, DCM, below DCM, and mesopelagic) were analyzed using one-way analysis of variances (ANOVAs) and a Tukey’s HSD test to perform multiple comparisons. Significant relationships were considered at α < 0.05. Normality and homoscedasticity were assessed with Q-Q plots and residual plots for each variable. Due to the non-homoscedasticity of the observed number of ASVs and the Chao index, these data were square-root-transformed to meet ANOVA assumptions.

Spearman’s rank correlation coefficients were employed to assess the correlations between individual ASVs abundance and the concentrations of inorganic N and the physicochemical parameters. Only samples from transect A were selected due to the lack of O_2_, turbidity, and fluorescence data in eight of the 12 samples from transect C. Low abundance ASVs (that do not appear more than two times in at least four samples) were excluded from this analysis to avoid low degrees of freedom. Correlations were obtained on a centered log ratio transformed ASV table (Gloor et al., 2017). Spearman’s rank correlation coefficients were also calculated between all ASVs using the ELSA pipeline (Xia et al., 2011, 2013). A network analysis for the significant correlations found between ASVs was created with the igraph R package (Csardi and Nepusz, 2006) and subcommunities/modules were found using the Louvain method (Blondel et al., 2008). A cut off of ρ > | 0.7|, with a *p*-value < 0.001, was imposed to consider the Spearman’s correlations significant. For both physicochemical and biological correlation analyses, only the samples from below DCM and mesopelagic were used to avoid the effect of auto-correlation among several variables in largely distinct water column sections.

All statistical analyses were conducted in the R environment (version 3.2.2. Copyright 2015, the R Foundation for Statistical Computing). Most plots were obtained with base R and the ggplot2 R package, while maps were created with “rnaturalearth,” “ggplot2,” “sf,” and “ggspatial” R packages, and edited with Gimp (v.2.8.14).

## Results

### Physicochemical Context

**FIGURE 2:**
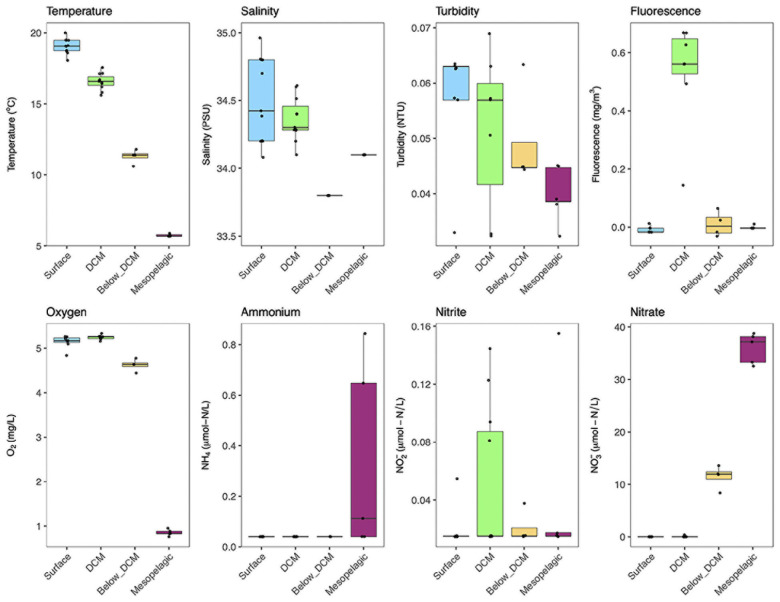
Physicochemical and dissolved inorganic nitrogen (DIN) conditions at the different sampling depths. Each sample is represented by one point. The boxes represent the first and third quartiles, with median value bisecting each box. The whiskers extend to the largest/smallest value, excluding outliers (data beyond 1.5× inter-quartile range).

The physicochemical parameters and inorganic N concentrations measured at the different depths are shown in Figure 2 (see Supplementary Figure 1 for the full CTD profiles). The temperature dropped consistently with depth, from mean 19°C at the surface to 6°C in the mesopelagic. Salinity was generally higher in samples from the surface and DCM, especially when compared to samples collected just below the DCM. The variability in salinity values was also considerably higher in surface and DCM samples. The studied region has very clear waters, with low turbidity levels across the different water column depths. The fluorescence levels peaked at the DCM while the other depth layers had near-zero values in most samples. Oxygen concentrations dropped from around 5.2 mg L^–1^ at the surface and the DCM to 4.6 mg L^–1^ below the DCM and 0.85 mg L^–1^ in the mesopelagic. Considering inorganic nitrogen, we measured NH_4_^+^, NO_2_^–^, and NO_3_^–^ concentrations at the different depths. Dissolved NH_4_^+^ concentrations were below the limit of quantification (0.04 μM) in almost all samples, with the exception of the mesopelagic, that presented average values of 0.34 μM. Nitrite (NO_2_^–^) concentrations presented high variability in most depths. Nonetheless, a peak in NO_2_^–^ concentrations was observed in samples collected at the DCM. Nitrate, on the other hand, accumulated in deeper samples, with concentrations in the mesopelagic (36 μM) and below DCM (12 μM) being three orders of magnitude higher than those found at the DCM (0.036 μM). In surface waters, NO_3_^–^ levels were always below the quantification limit (0.015 μM). Overall, the four different depths were clearly distinct based on the measured physicochemical parameters and dissolved inorganic nitrogen (DIN) concentrations (see Supplementary Figure 2 for a principal component analysis plot).

### Community Composition of Nitrifying Archaea and Bacteria

**FIGURE 3:**
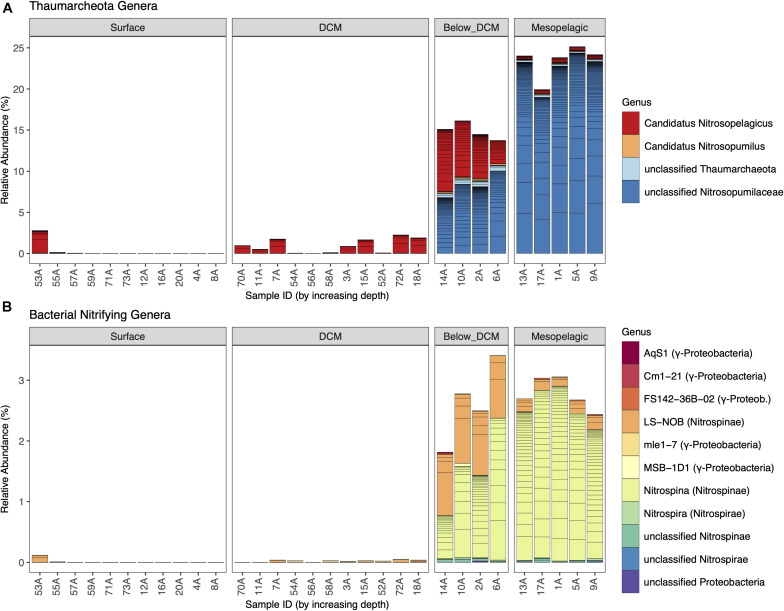
Thaumarcheotal **(A)** and nitrifying bacterial **(B)** community composition at the different depths. Each line of a stacked bar represents a unique amplicon sequence variant (ASV) and different genera are represented by different colors. Relative abundances of each ASV per sample were calculated by dividing the absolute abundance (counts) of each ASV by the sum of counts of all prokaryotic ASVs.

The relative abundances of thaumarchaeotal and nitrifying bacterial genera are shown in Figure 3 (overall prokaryotic community shown in Supplementary Figure 3). Considering Thaumarchaeota, a strong depth preference was observed. Despite the presence of the genus *Candidatus Nitrosopelagicus* in almost all depths sampled, the total relative abundance of thaumarcheotal genera increased from an average of less than 1% at the surface to around 15% below the DCM and almost 25% in the mesopelagic. This increment was mainly driven by the substantial increase in the relative abundance of unclassified Thaumarchaeota belonging to the Nitrosopumilaceae family, a well-known family of putative AOA. In fact, unclassified Nitrosopumilaceae dominated in the deeper layers, with more than 90% of the thaumarcheotal sequences in the mesopelagic and more than 50% below the DCM.

**FIGURE 4:**
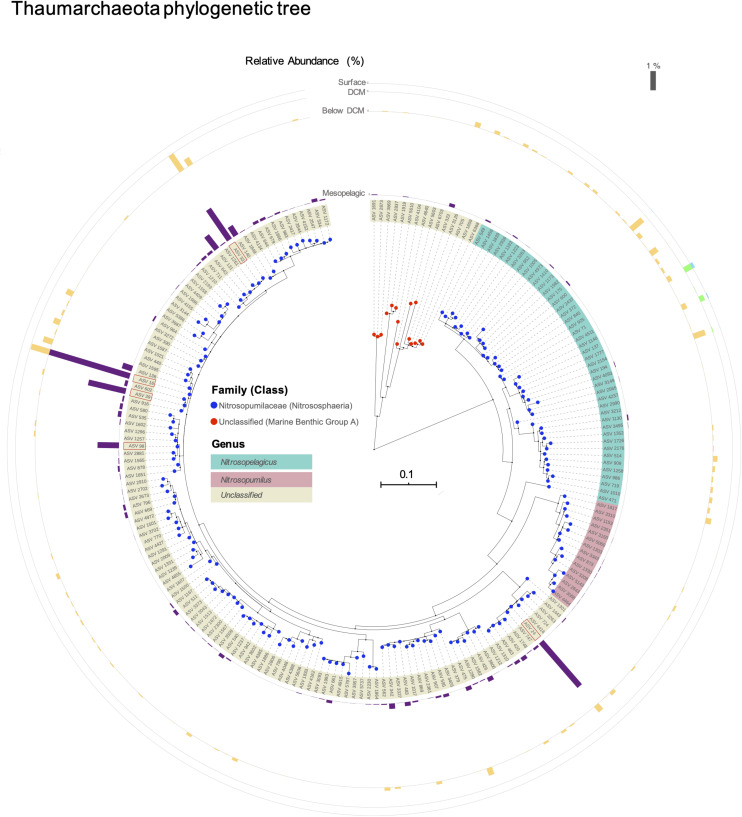
Neighbor-joining phylogenetic tree of all thaumarcheotal ASVs found in this study. Taxonomy was assigned with the silva v128 database. The bars in the outer circle represent the mean relative abundance of each ASV at the different depths in the water column (scale bar on the top right). The top 5 ASVs are highlighted with a red rectangle (ASV 33, ASV 16, ASV 39, ASV 98, and ASV 24). The tree was visualized and annotated using the iTOL software.

The phylogenetic distance between these unclassified ASVs and the other Thaumarchaeota present in this dataset is shown in Figure 4. The majority of unclassified ASVs at the genus level are closer to the *Candidatus Nitrosopumilus* genus than to the *Ca. Nitrosopelagicus*. However, four of the top five most abundant ASVs, namely ASV 16, ASV 33, ASV 39, and ASV 98, present a high genetic distance to both genera. To further explore these unknown classifications, we applied a different classifier and reference database to the sequence data. These alternative taxonomic classifications are shown in Table 2. The ambiguity persisted, i.e., the top five ASVs (with the exception of ASV 24, which had previously been found to be closer to *Ca. Nitrosopumilus*) could be assigned to either *Ca. Nitrosopumilus*, *Ca. Nitrosopelagicus*, or unclassified, depending on the classifier and classification algorithm used.

**TABLE 2:** Taxonomic assignments of the five most abundant thaumarchaeotal ASVs.

ASV	Classifier	Database	Class	Order	Family	Genus
ASV 16	DADA2-Bayesian	Silva	Nitrososphaeria	Nitrosopumilales	Nitrosopumilaceae	NA
		GTDB	NA	Nitrosopumilales	Nitrosopumilaceae	Nitrosopumilus
	IDTAXA	Silva	Nitrososphaeria	Nitrosopumilales	Nitrosopumilaceae	NA
		GTDB	Nitrososphaeria	Nitrososphaerales	Nitrosopumilaceae	Nitrosopelagicus
ASV 24	DADA2-Bayesian	Silva	Nitrososphaeria	Nitrosopumilales	Nitrosopumilaceae	NA
		GTDB	NA	Nitrosopumilales	Nitrosopumilaceae	Nitrosopumilus
	IDTAXA	Silva	Nitrososphaeria	Nitrosopumilales	Nitrosopumilaceae	NA
		GTDB	Nitrososphaeria	Nitrososphaerales	Nitrosopumilaceae	NA
ASV 39	DADA2-Bayesian	Silva	Nitrososphaeria	Nitrosopumilales	Nitrosopumilaceae	NA
		GTDB	NA	Nitrosopumilales	Nitrosopumilaceae	Nitrosopumilus
	IDTAXA	Silva	Nitrososphaeria	Nitrosopumilales	Nitrosopumilaceae	NA
		GTDB	Nitrososphaeria	Nitrososphaerales	Nitrosopumilaceae	Nitrosopelagicus
ASV 33	DADA2-Bayesian	Silva	Nitrososphaeria	Nitrosopumilales	Nitrosopumilaceae	NA
		GTDB	NA	Nitrosopumilales	Nitrosopumilaceae	Nitrosopumilus
	IDTAXA	Silva	Nitrososphaeria	Nitrosopumilales	Nitrosopumilaceae	NA
		GTDB	Nitrososphaeria	Nitrososphaerales	Nitrosopumilaceae	Nitrosopelagicus
ASV 98	DADA2-Bayesian	Silva	Nitrososphaeria	Nitrosopumilales	Nitrosopumilaceae	NA
		GTDB	NA	Nitrosopumilales	Nitrosopumilaceae	Nitrosopumilus
	IDTAXA	Silva	Nitrososphaeria	Nitrosopumilales	Nitrosopumilaceae	Nitrosopumilus
		GTDB	Nitrososphaeria	Nitrososphaerales	Nitrosopumilaceae	Nitrosopelagicus

When considering putative nitrifying bacteria (Figure 3B), these followed the same depth preference as Thaumarchaeota, although with substantially lower levels than their archaeal counterparts. Their maximum total prokaryotic community relative abundance was around 3%, below the DCM and in the mesopelagic. Concerning AOB, we identified the presence of four genera (AqS1, Cm1-21, FS142-36B-02, and MSB-1D1) and unclassified ASVs belonging to the Nitrosococcaceae family (Nitrosococcales order of γ-Proteobacteria) and the mle1-7 genus belonging to the Nitrosomonadaceae family (Betaproteobacteriales order of γ-Proteobacteria) at very low relative abundances (<0.05%). All other nitrifying bacteria identified were putative NOB, belonging to the Nitrospinae or Nitrospirae phyla. *Nitrospina* was the most abundant NOB genus, especially in the mesopelagic, representing around 90% of all putative nitrifying bacteria. Other important nitrite oxidizers, like *Nitrobacter*, were not found to be present in this dataset.

### Richness and Diversity of Nitrifying Archaea and Bacteria

**FIGURE 5:**
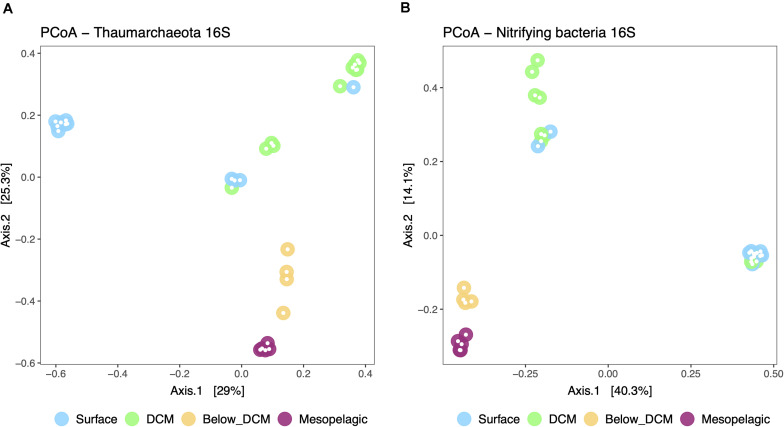
Principal coordinate analysis (PCoA) plot representing the β-diversity of the thaumarchaeotal **(A)** and nitrifying bacterial **(B)** community from the different depths in the water column. Sample dissimilarity and distance analysis were calculated using the Bray-Curtis dissimilarity index. The group dispersion was not significantly different across depths for the Thaumarcheotal community (permdisp, *p* = 0.256) but it was marginally different in the nitrifying bacterial community (permdisp, *p* = 0.016).

A principal coordinate analysis (PCoA) was performed to represent the β-diversity of nitrifier communities based on the calculated dissimilarities among samples (Figure 5). The dissimilarity among samples was based on the distribution of 196 thaumarchaeotal ASVs and 95 putative nitrifying bacterial ASVs, representing 98 and 92% of the total number of ASVs in each nitrifiers’ community, respectively. The first two principal coordinates represented 54.3 and 54.4% of the variation in thaumarcheotal and nitrifying bacterial communities, respectively. Thaumarchaeotal β-diversity (Figure 5A) was significantly affected by depth (PERMANOVA, *p* < 0.05). Samples from the mesopelagic and below DCM are generally similar to each other while the samples from the surface and the DCM cluster further apart. This is possibly explained by the shift observed from a *Ca. Nitrosopelagicus* dominated thaumarchaeotal community at the surface and DCM to a more diverse community below the DCM and in the mesopelagic. Nitrifying bacteria (AOB and NOB) β-diversity (Figure 5B) was also significantly affected by depth (PERMANOVA, *p* < 0.05) and presented the same mesopelagic/below DCM cluster, positioned further away from the surface and DCM samples. The drastic increase in Nitrospinae bacteria, especially *Nitrospina*, below the DCM and the mesopelagic possibly caused this shift. The significant depth effect in structuring the thaumarcheotal and nitrifying bacterial communities was also observed for the overall prokaryotic communities (Supplementary Figure 4).

**FIGURE 6:**
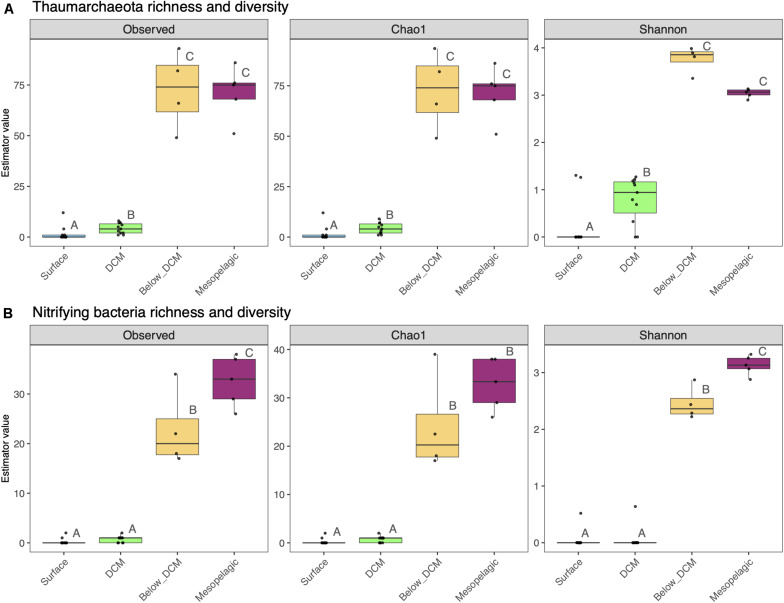
Species richness and α-diversity of thaumarchaeotal **(A)** and nitrifying bacterial **(B)** communities from the different depths in the water column. Each sample is represented by one point. The boxes represent the first and third quartiles, with median value bisecting each box. The whiskers extend to the largest/smallest value, excluding outliers (data beyond 1.5× inter-quartile range). Dissimilar letters denote a significant difference (one-way ANOVA, *p* < 0.05) between the depth layers.

The depth effect in species richness and α-diversity of putative nitrifying prokaryotes is displayed in Figure 6. Species richness of thaumarchaeota, estimated by the number of observed ASVs and the Chao index, increased significantly below the DCM and in the mesopelagic, when compared to the surface and DCM (one-way ANOVA, *p* < 0.05). Thaumarcheotal α-diversity, estimated through the Shannon index (H′), peaked in samples collected just below the DCM and it was significantly higher at this depth and in the mesopelagic than in surface and DCM samples (one-way ANOVA, *p* < 0.05). A similar pattern was observed for putative nitrifying bacteria as well as the overall prokaryotic community (Supplementary Figure 5), with an increase in species richness and diversity with depth. The highest values, however, were observed at the mesopelagic, not below the DCM, as it happened with the Thaumarchaeota.

### Environmental and Biological Interactions

**FIGURE 7:**
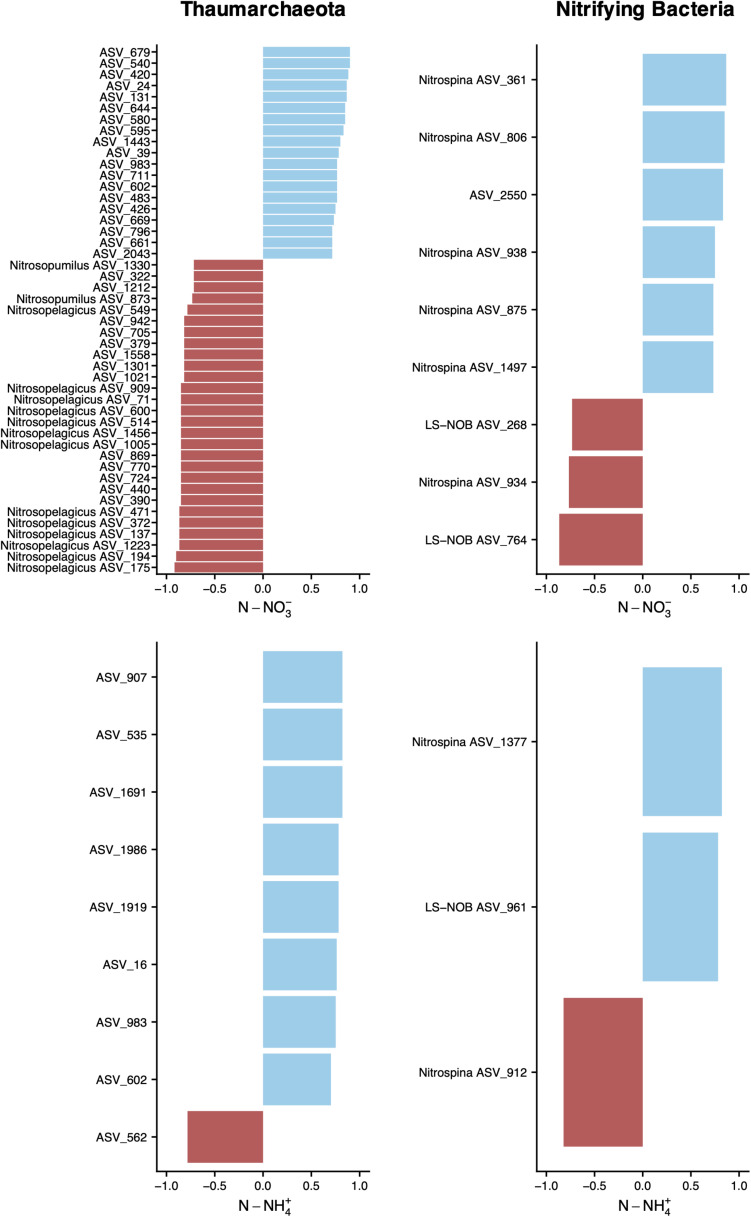
Relationship between dissolved inorganic N and ASV abundance. ASVs without a genus name in the vertical axis represent unclassified ASVs at the genus level. Correlations were assessed by Spearman’s rank coefficients (horizontal axis) for archaeal and nitrifying bacterial ASVs. Spearman’s ranks are shown for ρ > | 0.7|, with a *p*-value < 0.001 and were obtained on a centered log ratio transformed biological dataset, after selecting for ASVs that occur more than two times in at least four sampling sites of transect A. Only samples from below DCM and mesopelagic were included to avoid the auto-correlation effect among several variables caused by the sharp gradients across the different water column sections.

The abundance of putative nitrifying thaumarchaeota and bacteria was higher at below DCM and mesopelagic sampling sites, where concomitantly the concentrations of dissolved NH_4_^+^ and NO_3_^–^ were higher. To inspect the relationships between the abundance of thaumarchaeotal and nitrifying bacterial ASVs with the concentrations of DIN, we employed Spearman’s rank correlation coefficients (Figure 7). Nineteen unclassified thaumarchaeotal ASVs, all belonging to the Nitrosopumilaceae family, showed a significant positive relationship with NO_3_^–^ concentrations, while known AOA, mainly assigned to the *Ca. Nitrosopelagicus* genus, showed a negative relationship. Eight positive correlations and one negative were found between NH_4_^+^ and thaumarchaeotal ASVs, mostly with unclassified ASVs from the Nitrosopumilaceae family, but it’s worth noting that ASV 1691 lowest assignment was to phylum Thaumarchaeota, and ASV 1919 lowest assignment was to class Marine Benthic Group A.

Putative NOB identified as being associated with changes in DIN concentrations could be assigned either to *Nitrospina* sp. or LS-NOB, both in the family Nitrospinaceae, with the exception of ASV 2550, with the lowest taxonomic assignment at the phylum level (Nitrospinae). Further inspection of all correlations showed that ASVs assigned to LS-NOB predominantly exhibited a negative correlation with NO_3_^–^ concentrations and a positive correlation with NH_4_^+^, as shown in Figure 7. On the other hand, *Nitrospina* sp., in spite of showing predominantly positive correlations with DIN concentrations, also displayed two ASVs negatively correlated with N-NH_4_^+^ and N-NO_3_^–^ concentrations. Other bacterial groups of interest, such as AOB, could not be correlated with DIN concentration. ASVs assigned to families Nitrospiraceae and Nitrosomonadaceae only occurred in one or two sampling sites, making correlations unfeasible.

**FIGURE 8:**
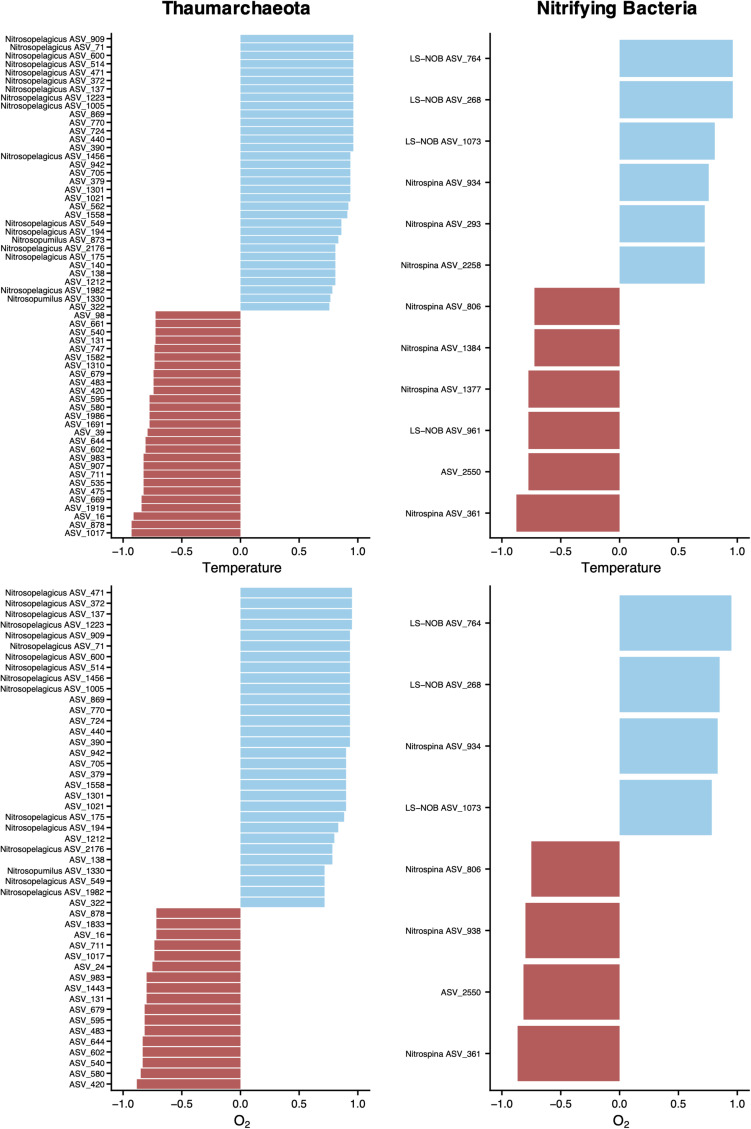
Relationship between physicochemical parameters and ASV abundance. ASVs without a genus name in the vertical axis represent unclassified ASVs at the genus level. Correlations were assessed by Spearman’s rank coefficients (horizontal axis) for archaeal and nitrifying bacterial ASVs. Spearman’s ranks are shown for ρ > | 0.7|, with a *p*-value < 0.001 and were obtained on a centered log ratio transformed biological dataset, after selecting for ASVs that occur more than two times in at least four sampling sites of transect A. Only samples from below DCM and mesopelagic were included to avoid the auto-correlation effect among several variables caused by the sharp gradients across the different water column sections.

To explore the interactions between putative nitrifying ASVs and the abiotic environment of the water column, we performed a Spearman’s rank analysis between the abundance of selected ASVs, temperature, and oxygen (Figure 8). The relationships between thaumarchaeotal ASVs and these variables showed that AOA assigned to the *Ca. Nitrosopelagicus*, *Ca. Nitrosopumilus*, and some unclassified genera correlated positively with temperature and O_2_. However, the negative correlations with temperature and O_2_ were found exclusively with unclassified thaumarchaeotal ASVs. When considering putative NOB, our correlation analysis showed significant positive and negative correlations, regardless of the taxonomic classification.

**FIGURE 9:**
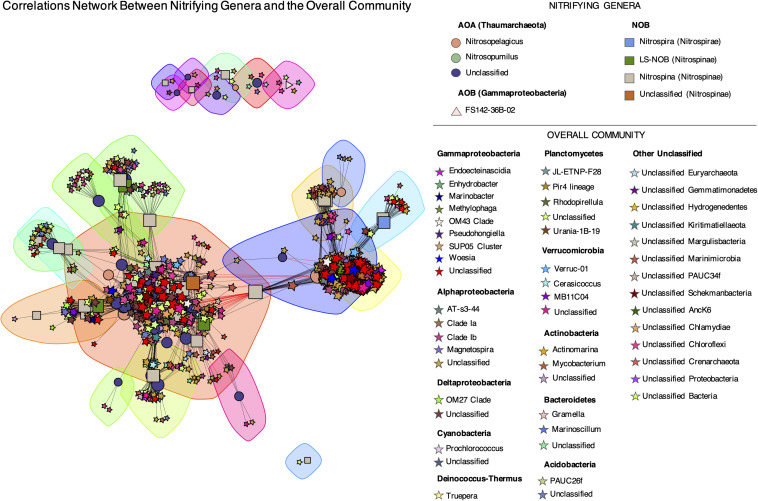
Network analysis of putative nitrifying ASVs and the overall community below the DCM and in the mesopelagic. Relationships were assessed by Spearman’s rank coefficients and only correlations with ρ > | 0.7|, *p*-value < 0.001, and degree > 0 are shown. Edge color indicates sign of correlation: negative (red) and positive (black). Node size is proportional to node degree (number of significant correlations). Node taxon is represented by different colors while the shapes represent functional guilds: AOA (circles), AOB (triangles), NOB (squares), and overall community (stars). Sub-communities are highlighted with shaded areas and were found using the Louvain method (Blondel et al., 2008). The network layout was obtained with the force-directed layout algorithm by Fruchterman and Reingold using the igraph R package (Csardi and Nepusz, 2006).

Quantitative associations within and between nitrifying ASVs and the overall microbiome present in below DCM and mesopelagic samples were evaluated through Spearman correlations and a corresponding network analysis (Figure 9). A total of 13230 significant correlations (edges) were found between 872 unique ASVs (717 non-nitrifying and 155 potential nitrifiers). A median number of 19 significant interactions per ASV was found across the network. The large majority of these interactions (99.5%) were positive correlations. By employing the Louvain method, 23 subcommunities/modules were found. It is worth noticing that AOA and NOB co-occur within several nodules and that all subcommunities included ASVs from different genera. No modules were found to be taxonomically or functionally exclusive. In fact, a large diversity of non-nitrifying taxa, from 20 different phyla, correlated strongly with nitrifying ASVs. Among those, unclassified Chloroflexi, Marinimicrobia, α- and γ-Proteobacteria had the highest number of correlations, with 5821 significant correlations in total and an average of 11.1–19.2 significant correlations/ASV (Supplementary Table 2). If unclassified genera are not considered, the non-nitrifying genera with the highest number of correlations with nitrifying ASVs were Rhodopirellula (Planctomycetes), Marinoscillum (Bacteroidetes), Pseudohongiella (γ-Proteobacteria), and Woeseia (γ-Proteobacteria), with 440 significant correlations in total and an average of 15.5–31.8 significant correlations/ASV (Supplementary Table 2).

## Discussion

In this study, we found extremely contrasting environments in the top 500 m of the water column, similarly to previous studies in the Pacific Ocean and other stratified water columns (Sun et al., 2019; Faull et al., 2020). In surface waters (3–5 m deep) and in the DCM (107–130 m deep), relatively high temperatures and oxygen levels were measured (Figure 2). The salinity and turbidity levels at these two depths suggest a well-mixed surface layer. Moving down the water column, below the DCM and in the mesopelagic, the environment was much colder and oxygen-poor, especially in the mesopelagic, where the O_2_ concentrations reached average levels of around 50 μM, still 10–100 times higher than levels found in oxygen minimum zones, such as the one located in the Eastern Tropical North Pacific (Faull et al., 2020). The DIN concentrations also showed a clear depth gradient. Surface and DCM waters were practically depleted of DIN (with the exception of NO_2_^–^ in the DCM), while below DCM and mesopelagic water masses accumulated high amounts of DIN, especially NO_3_^–^, the end-product of nitrification. In summary, two main contrasting environments were noted, a warmer O_2_-rich surface layer depleted in DIN and a colder O_2_-poor water below 150 m with high NO_3_^–^ concentrations.

This oceanographic split between surface/DCM and below DCM/mesopelagic was also evident in the community composition and β-diversity of putative nitrifying archaea and bacteria (Figures 3, 5). Thaumarchaeotal and nitrifying bacterial ASVs were rare in surface and DCM layers while relatively abundant (especially Thaumarchaeota) below the DCM and in the mesopelagic, as previously reported in this region as well as in microplankton communities from other regions, such as the Mediterranean Sea (Mincer et al., 2007; Santoro et al., 2010; Besseling et al., 2019; Mende et al., 2019). In deeper water columns, such as in the Marianas Trench, the increase in thaumarcheotal relative abundance is observed deeper than the mesopelagic (Zhong et al., 2020). In other regions, such as the northeastern South China Sea, thaumarcheotal abundance can remain homogeneously low throughout the water column, hypothetically due to an intense vertical mixing (Liu et al., 2017).

The exclusion of nitrifying microorganisms in nitrogen-limited sunlit layers is expected in mid-latitude oceans due to the high nitrogen assimilation capacity of dominating photosynthetic organisms (Harrison et al., 1996; Zakem et al., 2018) and to the indirect photoinhibition caused by reactive oxygen species (Tolar et al., 2016). This ecological competition also explains the overall low levels of all DIN species observed in the upper layers of the water column, with the exception of the NO_2_^–^ peak at the DCM (Figure 2). A NO_2_^–^ peak near the DCM has been previously observed in the Pacific (Shiozaki et al., 2016; Smith et al., 2016) and the Atlantic Ocean (Liu et al., 2018) and can be explained by differences in redox chemistry and cell size between ammonia- and nitrite-oxidizing organisms (Zakem et al., 2018). Smaller AOA grow faster and oxidize NH_4_^+^ to NO_2_^–^ at higher rates than larger NOB can oxidize NO_2_^–^ to NO_3_^–^ (Kuypers et al., 2018). This explanation is, in fact, consistent with the observation in our study of a subtle increase of AOA in the DCM without a concomitant increase in NOB.

Besides this broader split in community composition between shallower and deeper layers, we also observed some finer differences within each nitrifying group that may represent ecological and physiological differences. Most thaumarcheotal ASVs at the DCM were assigned to the *Ca. Nitrosopelagicus* genus (Figure 3). This genus has been shown to dominate thaumarcheotal sequences near the surface of the open ocean, probably due to its high genomic potential to survive in oligotrophic conditions (Santoro et al., 2015). With the increase in depth, the relative number of ASVs assigned to *Ca. Nitrosopelagicus* decreased and the majority of thaumarchaeotal sequences were unclassified at the genus level. In the North Pacific, the presence of diverse and abundant thaumarcheotal sequences that are unclassified at the genus level has been previously observed around the DCM and in the mesopelagic (Lund et al., 2012; Mende et al., 2019), which is probably explained by the high AOA diversity observed at these depths (Mincer et al., 2007; Santoro et al., 2010). The decrease in the abundance of *Ca. Nitrosopelagicus* can be partially caused by its growth temperature optimum of ∼22°C and completely halted growth in temperatures below 10°C (Santoro et al., 2015), such as the ones observed here in the mesopelagic.

Our phylogenetic analysis revealed that the unknown Thaumarchaeota that dominated the mesopelagic environment were closer to the *Ca. Nitrosopumilus* genus than to the *Ca. Nitrosopelagicus* (Figure 4). A depth-stratified population structure within phylogenetically related clades has been previously observed (Mende et al., 2019). A predominance of *Ca. Nitrosopumilus* over *Ca. Nitrosopelagicus* in deeper and colder waters could be attributed to the high thermal range observed for different *Ca. Nitrosopumilus* species (Walker et al., 2010; Bayer et al., 2019). The diverse unclassified sequences we found to be highly abundant in the mesopelagic may thus represent a new clade of *Ca. Nitrosopumilus* AOA or even a larger taxonomic division. However, due to the limited size of our 16S reads and the ambiguous classification from different databases (Table 2), we cannot confidently conclude that these unknown sequences would fall into the *Ca. Nitrosopumilus* or to a putative new genus. For a finer phylogenetic placement of these unknown sequences, larger DNA fragments need to be sequenced. Nevertheless, our data strongly suggests that an unknown group of closely related AOA is very well adapted to the conditions observed in the deeper layers of this water column.

When considering the abundance and depth distribution of NOB, we observed a sharp increase below the DCM and in the mesopelagic, marked by a strong dominance of Nitrospinae ASVs (Figure 3B). *Nitrospina*-like sequences dominated the NOB community across all samples, but they were particularly abundant below the DCM and in the mesopelagic, which is in accordance with previous studies from other regions showing a successful adaptation of *Nitrospina* to O_2_-poor mesopelagic waters (Zaikova et al., 2010; Füssel et al., 2012; Sun et al., 2019).

Much less is known about the α-diversity patterns of nitrifying organisms in the water column. In our study, the highest values of thaumarcheotal diversity were observed below the DCM and not in the mesopelagic, where their relative abundance was highest (Figures 6A, 3A). This result, together with the non-significant difference in species richness between the two depth layers, indicates that the community was more evenly distributed below the DCM while the mesopelagic was dominated by a fewer number of ASVs. When considering the richness and diversity of putative nitrifying bacteria, their highest values were observed in the mesopelagic (Figures 6B, 3B), despite the dominance of the *Nitrospina* genus. This result suggests that diversity within this NOB is particularly high, at least in O_2_-poor cold waters, which may contribute to their adaptability in this presumptive harsh environment for an obligate aerobe (Sun et al., 2019).

Our correlation analysis also revealed striking differences within thaumarcheotal ASVs between their abundances, DIN concentrations, and physicochemical parameters. Interestingly, only ASVs with unknown genus correlated positively with NO_3_^–^ (Figure 7), which suggests a crucial role of this group in regenerating NO_3_^–^ in this oligotrophic region. The importance of ammonia oxidation as the rate-limiting step of nitrification in the oceans is widely recognized (Lehtovirta-Morley, 2018), but our results suggest the existence of a particular thaumarcheotal group with stronger relevance than previously identified *Ca. Nitrosopelagicus* or *Ca. Nitrosopumilus* organisms, since the latter had non-significant or negative correlations with NO_3_^–^. The significant negative correlations of most unassigned ASVs with temperature and O_2_ (Figure 8) further indicate that these unknown AOA may be particularly well adapted to the colder O_2_-poor waters of the mesopelagic. This finding contrasts with general trends of increased AOA activity with temperature, previously documented for terrestrial, freshwater, and cultivated AOA (Lehtovirta-Morley, 2018). However, it is in agreement with previous work demonstrating high functional diversity and metabolic versatility among closely related thaumarchaeotal strains (Bayer et al., 2016).

Sometimes overlooked in environmental studies, biological interactions between different groups of nitrifying organisms play also a vital role in community structure (Jones and Hallin, 2019) and consequently, ecosystem functioning. Despite the oligotrophic nature of our study site, positive interactions dominated the correlations network between nitrifying ASVs (Figure 9). This observation indicates a strong co-occurrence between the microorganisms involved in nitrification, often reported in the literature (Daebeler et al., 2014; Bartelme et al., 2017; Jones and Hallin, 2019), and supports a theoretical hypothesis that a nutrient-limited environment would lead to stronger positive than negative interactions. Besides the interactions within nitrifying ASVs, we also observed a complex network of correlations between this group and diverse taxa of non-nitrifying prokaryotes (Figure 9). The highest degree of correlations was found for unclassified ASVs belonging to Chloroflexi, Marinimicrobia (formerly known as Marine Group A and SAR406), α- and γ-Proteobacteria (Supplementary Table 2). Both α- and γ-Proteobacteria are dominant classes of heterotrophic bacteria in the marine environment (Sanz-Sáez et al., 2020), while Chloroflexi organisms represent a diverse range of metabolisms, such as aerobic/anaerobic heterotrophy or anoxygenic photosynthesis, and are present in multiple habitats around the planet, from the human oral cavity to sponges or the deep sea (Bayer et al., 2018). Marinimicrobia, with no cultured representatives so far, is globally distributed in the oceans and appears to have a wide diversity of metabolic properties and syntrophic interactions (Hawley et al., 2017). A recent metagenomic and metatranscriptomic study showed that some Marinimicrobia lineages can participate in strong co-metabolic interactions within the nitrogen cycle, particularly by expressing the nitrous oxide (N_2_O) reductase gene (*nosZ*), which allows the coupling to thaumarchaeotal AOA that produce N_2_O as a byproduct of ammonia oxidation (Santoro et al., 2011; Hawley et al., 2017). This potential link to AOA may contribute to explain the high relative abundance of unclassified Marinimicrobia observed in the below DCM and mesopelagic samples (Supplementary Figure 3). In our study, the network community modules found were taxonomically and functionally diverse. All subcommunities found included several AOA and NOB with different taxonomic assignments. Tight correlations between some specific nitrifying groups, such as AOA and Nitrospina species in open ocean waters have been previously documented (Mincer et al., 2007; Santoro et al., 2010), but a cosmopolitan interaction, such as the one observed in our study is seldom observed, probably because only specific groups are targeted in most studies.

Overall, the results from this study show that a diverse and potentially unknown group of thaumarchaeotal ASVs are crucial for NO_3_^–^ production below the euphotic zone in the subtropical North Pacific. Moreover, it indicates that this group is composed of organisms well-adapted to the colder and O_2_-poor waters of the mesopelagic, where they thrive and become critical for N recycling. This study further demonstrates that, despite their dominance below the DCM and in the mesopelagic, this group is tightly linked to multiple NOB and that diverse subcommunities are present in the oligotrophic deep ocean. Future research addressing this large group of unclassified Thaumarchaeotal organisms will certainly contribute to improve our understanding about the role of AOA in recycling inorganic nitrogen in the remote oligotrophic ocean.

## Data Availability Statement

The datasets presented in this study can be found in online repositories. The web links and accession numbers can be found below: https://www.ebi.ac.uk/ena, PRJEB32783 (16S amplicon sequencing data); and https://doi.pangaea.de/10.1594/PANGAEA.903405 (CTD dataset).

## Author Contributions

CM conceived and designed the field sampling for this study. JG, MT, and CM performed the field sampling and data collection. MT performed the sample preparation for sequencing. MS, EL, MB, and MT analyzed the sequencing data. AO and JG analyzed the nutrient data. MS, EL, MB, and CM wrote the manuscript. All authors approved the final submitted manuscript.

## Conflict of Interest

The authors declare that the research was conducted in the absence of any commercial or financial relationships that could be construed as a potential conflict of interest.

## Footnotes

1 https://doi.pangaea.de/10.1594/PANGAEA.903405

## Supplementary Material

Supplementary Figure 1 Water column profiles of temperature, salinity, turbidity, fluorescence, and oxygen from casts performed along with sample collection.

Supplementary Figure 2 Principal component analysis of environmental parameters between samples collected at the different depths in the water column.

Supplementary Figure 3 Prokaryotic community composition at the different depths of the water column.

Supplementary Figure 4 Principal coordinate analysis (PCoA) plot representing the β-diversity of the overall prokaryotic community from the different depths in the water column.

Supplementary Figure 5 Species richness and α-diversity of prokaryotic communities from the different depths in the water column.

Supplementary Table 1 Summary of sequence processing in collected samples.

Supplementary Table 2 Taxonomic affiliation of non-nitrifying ASVs co-occurring with nitrifying ASVs.
