# Supplementary material for: Depth Profile of Nitrifying Archaeal and Bacterial Communities in the Remote Oligotrophic Waters of the North Pacific
Source: Front Microbiol. 2021 Feb 23;12:624071. doi: 10.3389/fmicb.2021.624071 (PMC7959781; doi:10.3389/fmicb.2021.624071)
Supplement: Supplementary Figure 1 — Water column profiles of temperature, salinity, turbidity, fluorescence, and oxygen from casts performed along with sample collection. [file Data_Sheet_1.PDF]

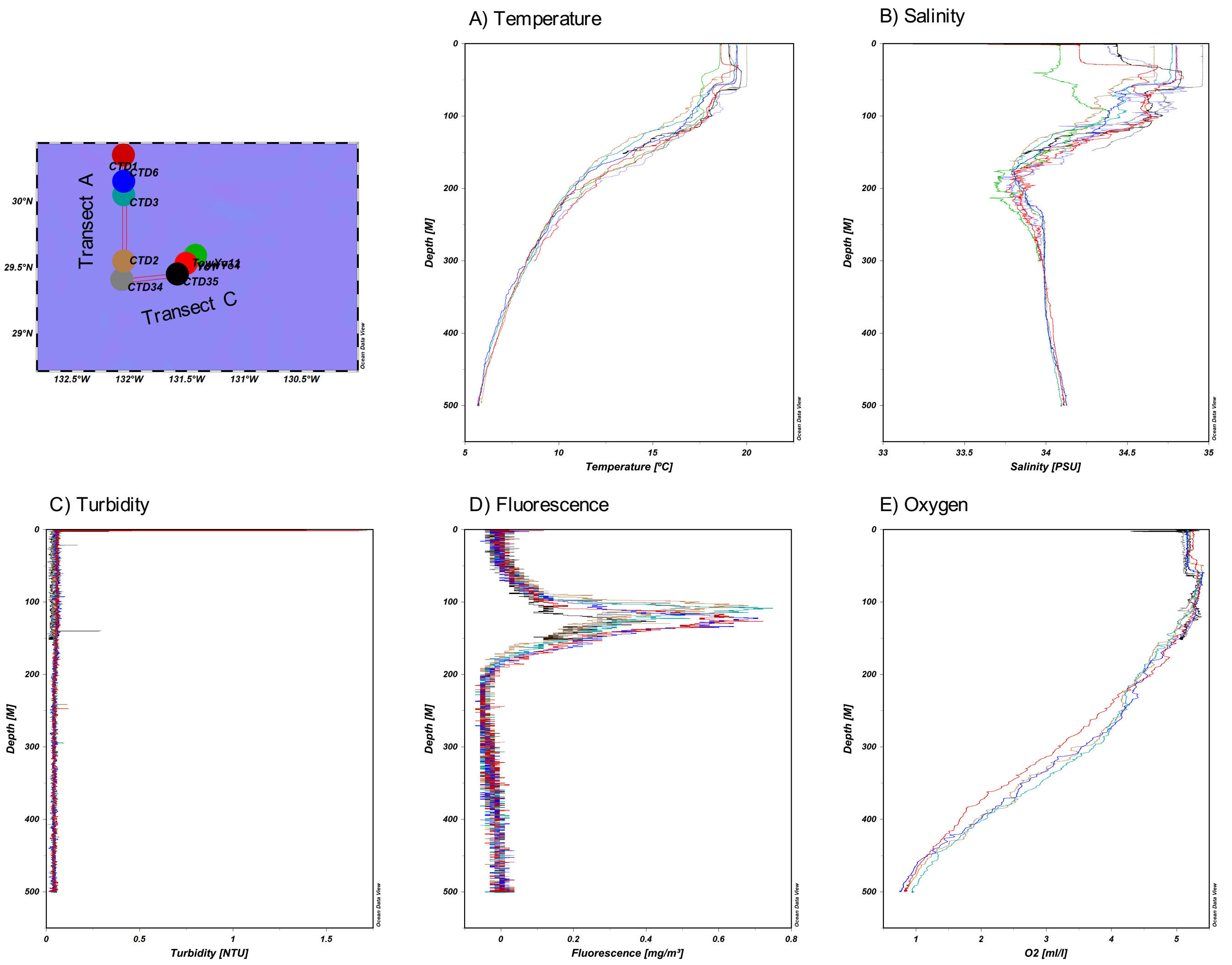


**Figure S1**. Water column profiles of temperature, salinity, turbidity, fluorescence, and oxygen from casts performed along with sample collection. Different colors correspond to different casts performed at the locations shown in the map (top left corner).
