# Supplementary material for: Depth Profile of Nitrifying Archaeal and Bacterial Communities in the Remote Oligotrophic Waters of the North Pacific
Source: Front Microbiol. 2021 Feb 23;12:624071. doi: 10.3389/fmicb.2021.624071 (PMC7959781; doi:10.3389/fmicb.2021.624071)
Supplement: Supplementary Figure 2 — Principal component analysis of environmental parameters between samples collected at the different depths in the water column. [file Data_Sheet_2.PDF]

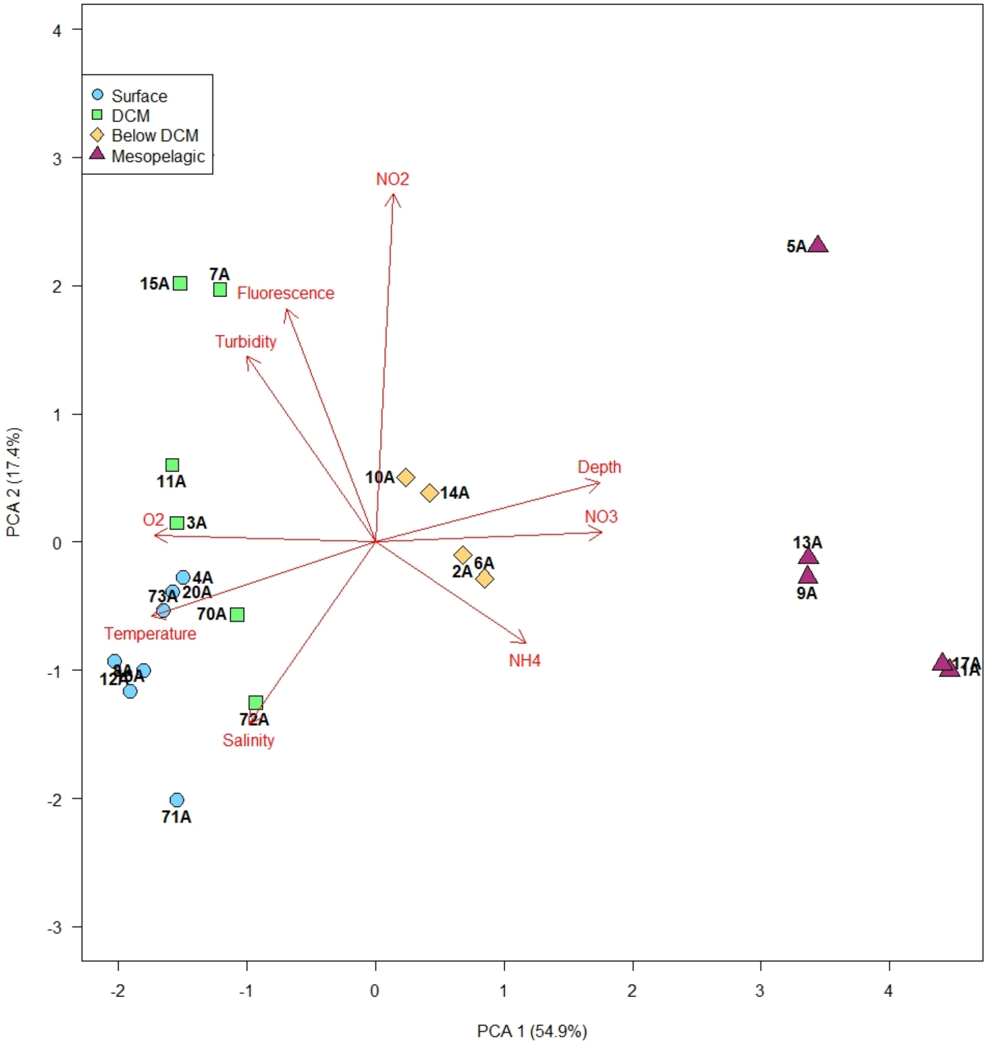

Figure S2. Principal component analysis of environmental parameters between samples collected at the different depths in the water column.
