# Supplementary material for: Depth Profile of Nitrifying Archaeal and Bacterial Communities in the Remote Oligotrophic Waters of the North Pacific
Source: Front Microbiol. 2021 Feb 23;12:624071. doi: 10.3389/fmicb.2021.624071 (PMC7959781; doi:10.3389/fmicb.2021.624071)
Supplement: Supplementary Figure 3 — Prokaryotic community composition at the different depths of the water column. [file Data_Sheet_3.PDF]

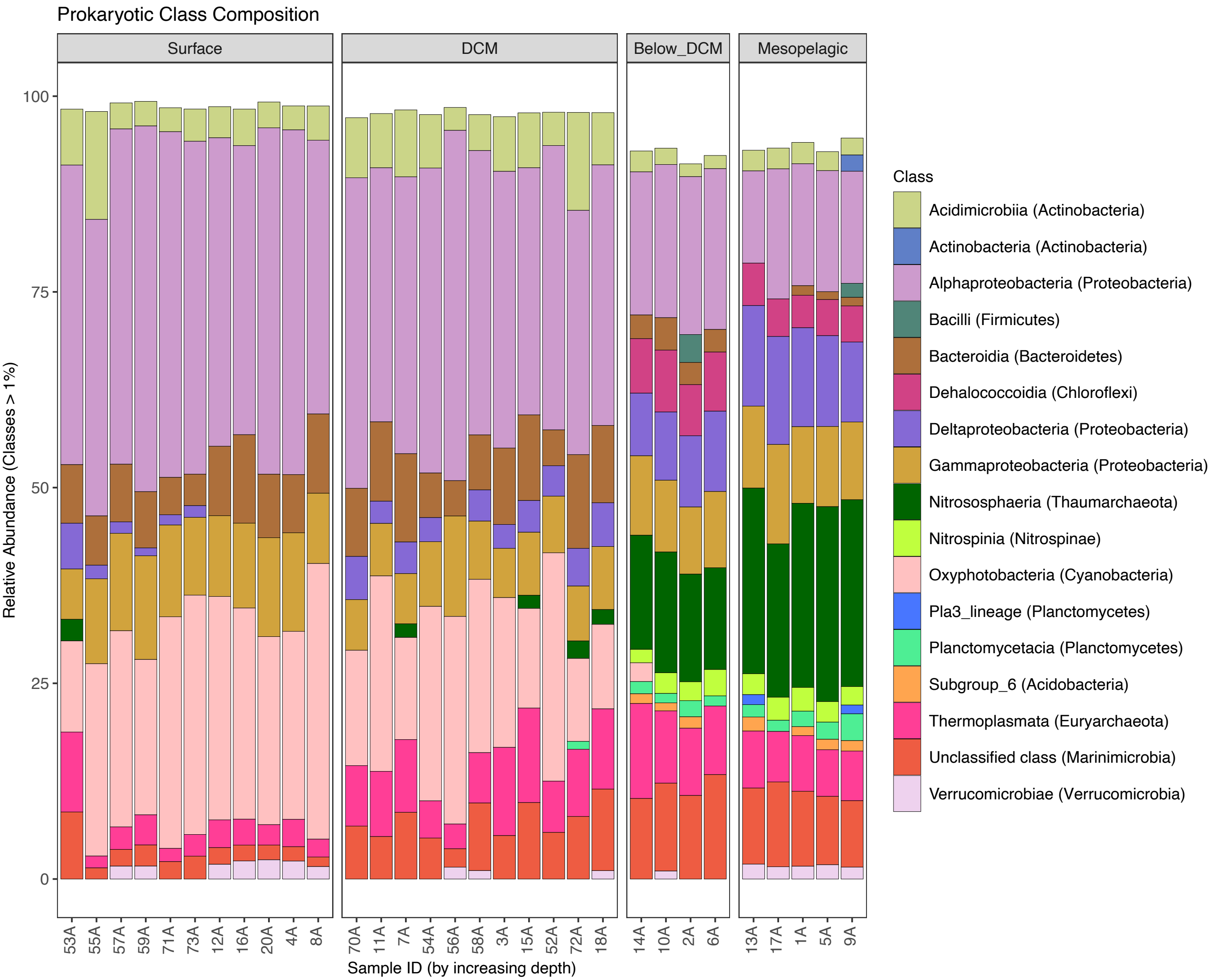

Figure S3. Prokaryotic community composition at the different depths of the water column. Only classes with relative abundance higher than 1% are shown. For each class, the corresponding phylum is shown in parenthesis in the legend.
