# Supplementary material for: Depth Profile of Nitrifying Archaeal and Bacterial Communities in the Remote Oligotrophic Waters of the North Pacific
Source: Front Microbiol. 2021 Feb 23;12:624071. doi: 10.3389/fmicb.2021.624071 (PMC7959781; doi:10.3389/fmicb.2021.624071)
Supplement: Supplementary Figure 4 — Principal coordinate analysis (PCoA) plot representing the β-diversity of the overall prokaryotic community from the different depths in the water column. [file Data_Sheet_4.PDF]

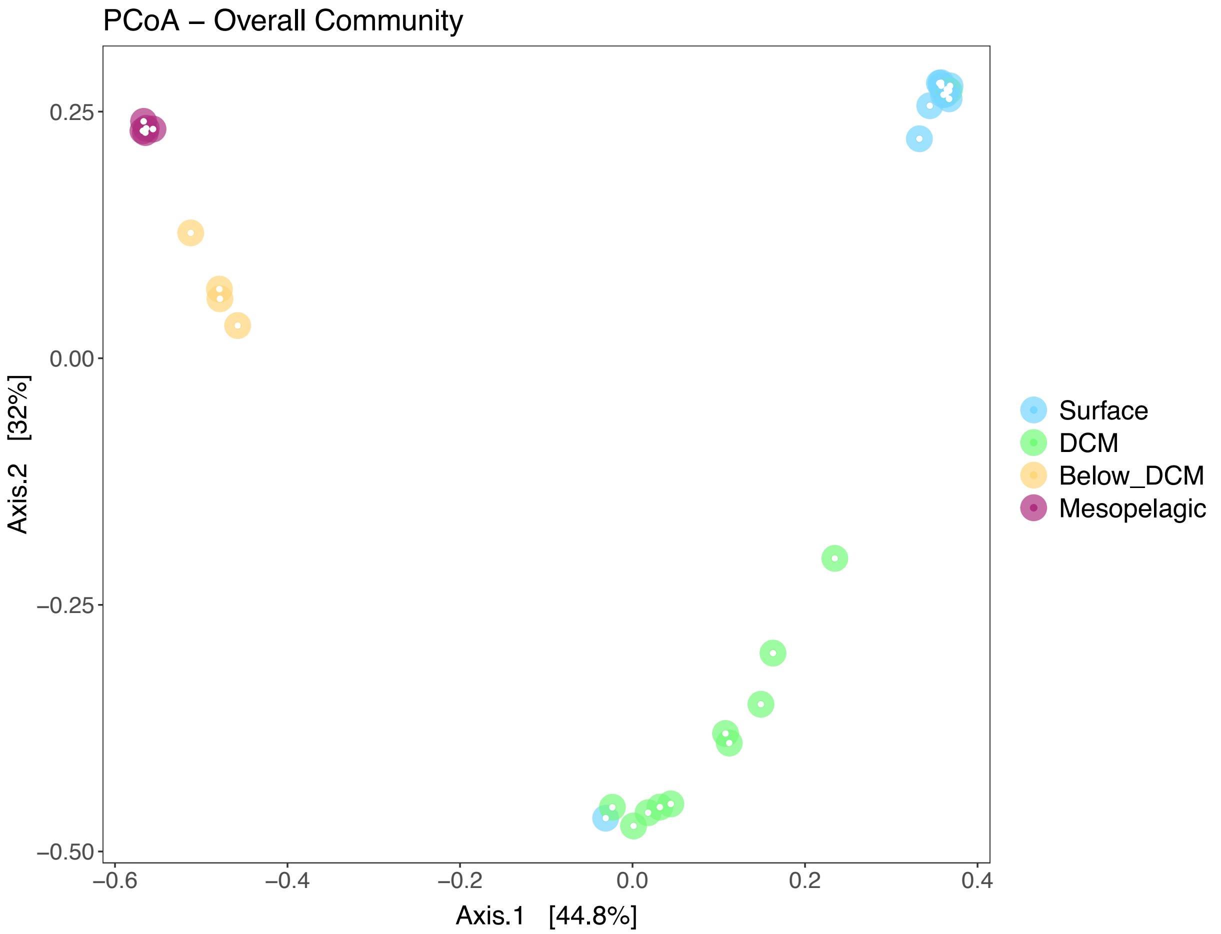

Figure S4. Principal coordinate analysis (PCoA) plot representing the β-diversity of the overall prokaryotic community from the different depths in the water column. Sample dissimilarity and distance analysis were calculated using the Bray−Curtis dissimilarity index on a rarefied abundance table.
