# Supplementary material for: Depth Profile of Nitrifying Archaeal and Bacterial Communities in the Remote Oligotrophic Waters of the North Pacific
Source: Front Microbiol. 2021 Feb 23;12:624071. doi: 10.3389/fmicb.2021.624071 (PMC7959781; doi:10.3389/fmicb.2021.624071)
Supplement: Supplementary Figure 5 — Species richness and α-diversity of prokaryotic communities from the different depths in the water column. [file Data_Sheet_5.PDF]

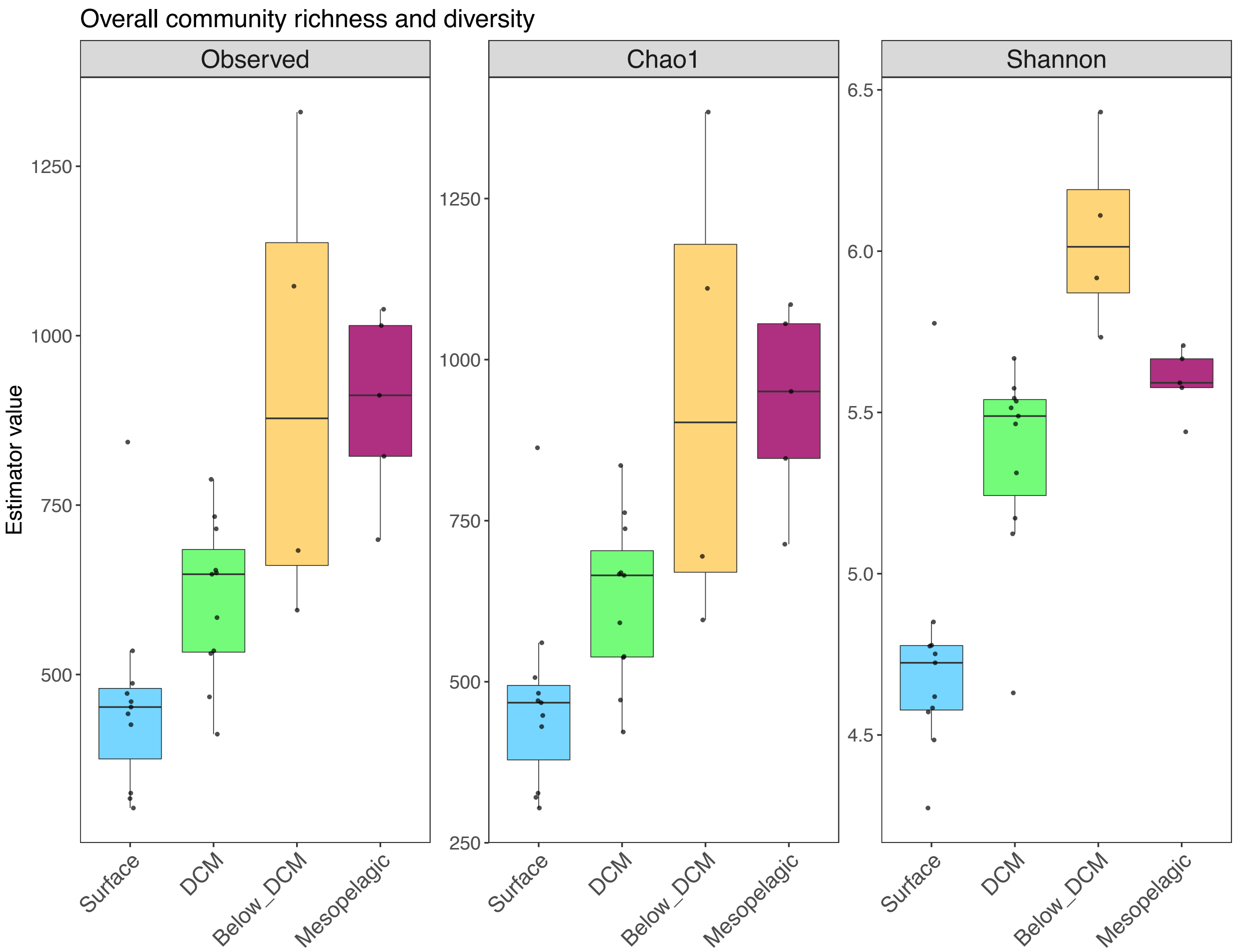

Figure S5. Species richness and α-diversity of prokaryotic communities from the different depths in the water column. Each sample is represented by one point. The boxes represent the first and third quartiles, with median value bisecting each box. The whiskers extend to the largest/smallest value, excluding outliers (data beyond 1.5 x inter-quartile range). The richness and diversity indexes were calculated from a rarefied abundance table.
